# (*Z*)-Ethyl 3-[bis­(4-chloro-3-ethyl-1-methyl-1*H*-pyrazol-5-yl­carbon­yl)amino]-3-(4-chloro­phen­yl)-2-cyano­propanoate

**DOI:** 10.1107/S1600536809008988

**Published:** 2009-03-28

**Authors:** Dehua Zhang, Xiaoyan Zhang, Lijuan Guo

**Affiliations:** a Department of Chemistry and Environmental Engineering, Hubei Normal University, Huangshi 435002, People’s Republic of China; b School of Mathematics and Physics, Huangshi Institute of Technology, Huangshi 435003, People’s Republic of China; c Department of Chemistry, Changsha Medical University, Changsha 410219, People’s Republic of China

## Abstract

The title compound, C_26_H_25_Cl_3_N_6_O_4_, was prepared by the reaction of (*Z*)-ethyl 3-amino-3-(4-chloro­phen­yl)-2-cyano­acrylate and 4-chloro-3-ethyl-1-methyl-1*H*-pyrazole-5-carbonyl chloride. The dihedral angles between the chloro­benzene and the two pyrazole rings are 59.8 (2) and 33.3 (2)°. The two pyrazole rings are oriented to each other at a dihedral angle of 84.7 (2)°. The crystal packing is governed by inter­molecular C—H⋯O inter­actions, resulting in a three-dimensional network. The ethyl groups are disordered over two positions, with site-occupancy factors of 0.71/0.29 and 0.51/0.49.

## Related literature

Several novel acrylate compounds are useful as inhibitors of *Pyricularia oryzae*, *Rhizoctonia solani*, *Botrytis cinerea* and *Gibberella zeae*; see: Heller *et al.* (2004 ▶); Creagh & Hubbell (1992 ▶); Ibers & Hamilton (1964 ▶).
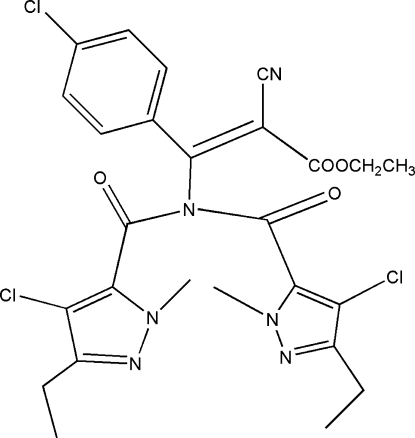


## Experimental

### 

#### Crystal data

C_26_H_25_Cl_3_N_6_O_4_
*M* *_r_* = 591.87
Monoclinic, 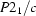

*a* = 10.7277 (3) Å
*b* = 16.1476 (5) Å
*c* = 17.3109 (5) Å
β = 107.671 (2)°
*V* = 2857.21 (14) Å^3^
*Z* = 4
Mo *K*α radiation
μ = 0.36 mm^−1^
*T* = 298 K
0.20 × 0.10 × 0.10 mm

#### Data collection

Bruker SMART CCD area-detector diffractometer
Absorption correction: none
18010 measured reflections
5600 independent reflections
3595 reflections with *I* > 2σ(*I*)
*R*_int_ = 0.045

#### Refinement

*R*[*F* ^2^ > 2σ(*F* ^2^)] = 0.065
*wR*(*F* ^2^) = 0.170
*S* = 1.02
5600 reflections
397 parameters
12 restraints
H-atom parameters constrained
Δρ_max_ = 0.33 e Å^−3^
Δρ_min_ = −0.21 e Å^−3^

### 

Data collection: *SMART* (Bruker, 1997 ▶); cell refinement: *SAINT* (Bruker, 1997 ▶); data reduction: *SAINT*; program(s) used to solve structure: *SHELXS97* (Sheldrick, 2008 ▶); program(s) used to refine structure: *SHELXL97* (Sheldrick, 2008 ▶); molecular graphics: *SHELXTL* (Sheldrick, 2008 ▶); software used to prepare material for publication: *SHELXTL*.

## Supplementary Material

Crystal structure: contains datablocks I, global. DOI: 10.1107/S1600536809008988/bq2113sup1.cif

Structure factors: contains datablocks I. DOI: 10.1107/S1600536809008988/bq2113Isup2.hkl

Additional supplementary materials:  crystallographic information; 3D view; checkCIF report

## Figures and Tables

**Table 1:** Hydrogen-bond geometry (Å, °)

*D*—H⋯*A*	*D*—H	H⋯*A*	*D*⋯*A*	*D*—H⋯*A*
C13—H13⋯O2^i^	0.93	2.59	3.420 (5)	149

Symmetry code: (i) 
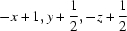
.

**Fig. 1.**
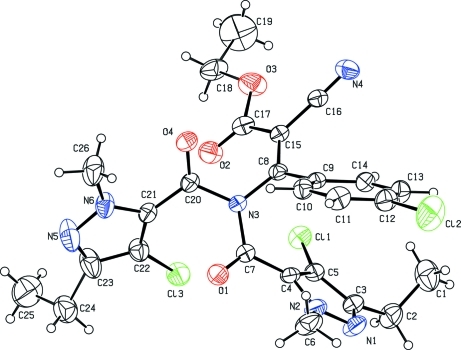
The molecular structure of the title compound, showing 30% probability displacement ellipsoids and the atom-numbering scheme. Only the higher occupied of the disordered sites is shown.

**Fig. 2.**
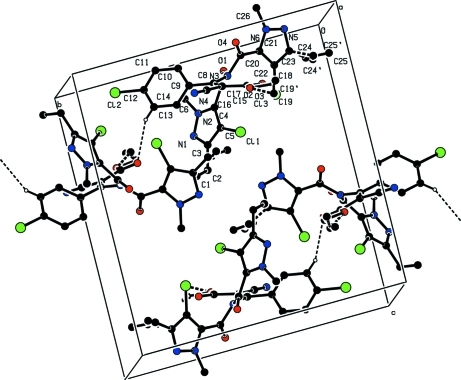
The packing of the title compound, viewed down the c-axis.

## supplementary crystallographic information

### Comment

Recently, 2-cyanoacrylates have been in widespread used as agrochemicals because of their unique mechanism of action and good environmental profiles. The title compound is useful as an inhibitor of Pyricularia oryzae, Rhizoctonia solani, Botrytis cinerea and Gibberella zeae (Heller *et al.*, 2004; Ibers & Hamilton, 1964). In the title compound (Fig. 1), all bond lengths and angles are unexceptional. The planar chlorobenzene ring is approximately perpendicular to one of the pyrazole ring with a dihedral angle of 59.9 (2)°. The dihedral angle between this chlorobenzene ring and the other pyrazole rings is 33.3 (12)°. The dihedral angle between the two pyrazole rings is 84.7 (2)°. The crystal packing is governed by C—H···O intermolecular interactions resulting in a three-dimensional network (Table 1.). The ethyl groups are disordered over two positions, with site-occupancy factors of 0.71/0.29 and 0.51/0.49.

### Experimental

To a solution of (*Z*)-ethyl 3-amino-3-(4-chlorophenyl)-2-cyanoacrylate (1.25 g, 0.0050 mol) in C_1_H_2_Cl_2_(18 ml), 4-chloro-3-ethyl-1-methyl- 1*H*-pyrazole-5-carbonyl chloride (3.11 g, 0.015 mol) was added. Subsequently, Et_3_N_1_(1.52 g, 0.015 mol) was dropped into the solution under stirring. The reaction mixture was then heated to reflux, stirred for 4 h. Subsequently, it was cooled to room temperature. The reaction solution was filtered off and some white solid was separated. The organic phase was washed with water and then dried over Na_2_S_1_O_4_. After removal of the solvent, a brown dope was obtained. After column chromatogram using ethylacetate/light petroleum (1:6) as the eluent, small single crystals were grown from a solution of ethyl acetate/petroleum ether(3:1) after 45 days, at room temperature.

### Refinement

Methyl H atoms were placed in calculated positions with C—H=0.96Å and the torsion angle was refined to fit the electron density; thermal parameters were refined as *U*_iso_(H)=1.5*U*_eq_(C). Other H atoms were placed in calculated positions with C—H =0.96Å (methylene C—H) and 0.93Å (aromatic C—H), and refined in riding mode, with *U*_iso_(H)=1.2*U*_eq_(C).

### Crystal data

**Table:**

C_26_H_25_Cl_3_N_6_O_4_	*F*(000) = 1224
*M**_r_* = 591.87	*D*_x_ = 1.376 Mg m^−^^3^
Monoclinic, *P*2_1_/*c*	Mo *K*α radiation, λ = 0.71073 Å
Hall symbol: -P 2ybc	Cell parameters from 2499 reflections
*a* = 10.7277 (3) Å	θ = 2.4–20.2°
*b* = 16.1476 (5) Å	µ = 0.36 mm^−^^1^
*c* = 17.3109 (5) Å	*T* = 298 K
β = 107.671 (2)°	Block, colorless
*V* = 2857.21 (14) Å^3^	0.20 × 0.10 × 0.10 mm
*Z* = 4	

### Data collection

**Table:**

Bruker SMART CCD area-detector diffractometer	3595 reflections with *I* > 2σ(*I*)
Radiation source: fine-focus sealed tube	*R*_int_ = 0.045
graphite	θ_max_ = 26.0°, θ_min_ = 1.8°
φ and ω scans	*h* = −13→13
18010 measured reflections	*k* = −14→19
5600 independent reflections	*l* = −19→21

### Refinement

**Table:**

Refinement on *F*^2^	Primary atom site location: structure-invariant direct methods
Least-squares matrix: full	Secondary atom site location: difference Fourier map
*R*[*F*^2^ > 2σ(*F*^2^)] = 0.065	Hydrogen site location: inferred from neighbouring sites
*wR*(*F*^2^) = 0.170	H-atom parameters constrained
*S* = 1.02	*w* = 1/[σ^2^(*F*_o_^2^) + (0.0809*P*)^2^ + 0.6207*P*] where *P* = (*F*_o_^2^ + 2*F*_c_^2^)/3
5600 reflections	(Δ/σ)_max_ < 0.001
397 parameters	Δρ_max_ = 0.33 e Å^−^^3^
12 restraints	Δρ_min_ = −0.21 e Å^−^^3^

### Special details

**Table:**

Geometry. All e.s.d.'s (except the e.s.d. in the dihedral angle between two l.s. planes) are estimated using the full covariance matrix. The cell e.s.d.'s are taken into account individually in the estimation of e.s.d.'s in distances, angles and torsion angles; correlations between e.s.d.'s in cell parameters are only used when they are defined by crystal symmetry. An approximate (isotropic) treatment of cell e.s.d.'s is used for estimating e.s.d.'s involving l.s. planes.
Refinement. Refinement of *F*^2^ against ALL reflections. The weighted *R*-factor *wR* and goodness of fit *S* are based on *F*^2^, conventional *R*-factors *R* are based on *F*, with *F* set to zero for negative *F*^2^. The threshold expression of *F*^2^ > σ(*F*^2^) is used only for calculating *R*-factors(gt) *etc*. and is not relevant to the choice of reflections for refinement. *R*-factors based on *F*^2^ are statistically about twice as large as those based on *F*, and *R*- factors based on ALL data will be even larger.

### Fractional atomic coordinates and isotropic or equivalent isotropic displacement parameters (Å^2^)

**Table:**

	*x*	*y*	*z*	*U*_iso_*/*U*_eq_	Occ. (<1)
C1	0.5605 (5)	1.1026 (3)	0.0809 (3)	0.1016 (16)	
H1A	0.4925	1.0742	0.0957	0.152*	
H1B	0.5308	1.1155	0.0241	0.152*	
H1C	0.5823	1.1529	0.1116	0.152*	
C2	0.6797 (4)	1.0481 (3)	0.0986 (2)	0.0819 (12)	
H2A	0.6586	0.9991	0.0647	0.098*	
H2B	0.7485	1.0777	0.0846	0.098*	
C3	0.7296 (4)	1.0218 (2)	0.1857 (2)	0.0600 (9)	
C4	0.7612 (3)	0.9620 (2)	0.30584 (19)	0.0464 (8)	
C5	0.6770 (3)	0.9653 (2)	0.22757 (19)	0.0493 (8)	
C6	0.9700 (4)	1.0424 (3)	0.3756 (3)	0.0843 (13)	
H6A	0.9397	1.0573	0.4205	0.126*	
H6B	1.0117	1.0893	0.3598	0.126*	
H6C	1.0316	0.9977	0.3913	0.126*	
C7	0.7665 (3)	0.91302 (19)	0.37875 (18)	0.0446 (7)	
C8	0.5482 (3)	0.96134 (19)	0.37330 (17)	0.0416 (7)	
C9	0.6001 (3)	1.0460 (2)	0.39174 (18)	0.0434 (7)	
C10	0.6964 (3)	1.0615 (2)	0.46510 (19)	0.0518 (8)	
H10	0.7256	1.0188	0.5023	0.062*	
C11	0.7483 (4)	1.1396 (3)	0.4827 (2)	0.0675 (10)	
H11	0.8112	1.1505	0.5320	0.081*	
C12	0.7058 (4)	1.2011 (2)	0.4263 (3)	0.0680 (11)	
C13	0.6112 (4)	1.1884 (2)	0.3540 (2)	0.0641 (10)	
H13	0.5830	1.2315	0.3173	0.077*	
C14	0.5585 (3)	1.1101 (2)	0.3366 (2)	0.0508 (8)	
H14	0.4946	1.1003	0.2875	0.061*	
C15	0.4182 (3)	0.9445 (2)	0.34745 (18)	0.0452 (7)	
C16	0.3269 (3)	1.0107 (2)	0.3428 (2)	0.0504 (8)	
C17	0.3619 (3)	0.8606 (3)	0.3228 (2)	0.0543 (9)	
C20	0.6330 (3)	0.8337 (2)	0.44703 (19)	0.0479 (8)	
C21	0.7060 (3)	0.7572 (2)	0.44792 (19)	0.0491 (8)	
C22	0.7445 (4)	0.7138 (2)	0.3903 (2)	0.0604 (9)	
C23	0.8073 (5)	0.6430 (3)	0.4273 (3)	0.0923 (14)	
C18	0.1713 (16)	0.7798 (7)	0.2921 (11)	0.077 (5)	0.51
H18A	0.2076	0.7451	0.2585	0.093*	0.51
H18B	0.1817	0.7522	0.3435	0.093*	0.51
C19	0.0337 (14)	0.7994 (15)	0.2510 (13)	0.159 (9)	0.51
H19A	−0.0021	0.8289	0.2876	0.238*	0.51
H19B	−0.0145	0.7491	0.2343	0.238*	0.51
H19C	0.0275	0.8332	0.2044	0.238*	0.51
C24	0.8921 (8)	0.5836 (4)	0.3948 (5)	0.089 (2)	0.71
H24A	0.8987	0.6030	0.3431	0.107*	0.71
H24B	0.9794	0.5793	0.4327	0.107*	0.71
C25	0.8247 (9)	0.5025 (4)	0.3851 (6)	0.127 (3)	0.71
H25A	0.8184	0.4845	0.4366	0.191*	0.71
H25B	0.8735	0.4626	0.3650	0.191*	0.71
H25C	0.7385	0.5079	0.3474	0.191*	0.71
C26	0.7327 (5)	0.7275 (3)	0.5962 (2)	0.0921 (14)	
H26A	0.6420	0.7356	0.5914	0.138*	
H26B	0.7811	0.7764	0.6186	0.138*	
H26C	0.7660	0.6812	0.6312	0.138*	
Cl1	0.53576 (9)	0.91015 (6)	0.18601 (5)	0.0620 (3)	
Cl2	0.77506 (16)	1.29995 (8)	0.44793 (10)	0.1232 (6)	
Cl3	0.71509 (10)	0.73843 (6)	0.29021 (5)	0.0700 (3)	
N1	0.8404 (3)	1.0541 (2)	0.23522 (19)	0.0693 (9)	
N2	0.8587 (3)	1.01616 (19)	0.30722 (17)	0.0596 (8)	
N3	0.6432 (2)	0.89762 (15)	0.39096 (14)	0.0414 (6)	
N4	0.2531 (3)	1.0622 (2)	0.3401 (2)	0.0723 (9)	
N5	0.8081 (4)	0.6418 (2)	0.5053 (2)	0.0992 (13)	
N6	0.7463 (3)	0.71124 (19)	0.51636 (17)	0.0667 (8)	
O1	0.8667 (2)	0.88843 (15)	0.42578 (14)	0.0596 (6)	
O2	0.4232 (2)	0.79961 (16)	0.31937 (17)	0.0697 (7)	
O3	0.2329 (2)	0.86236 (17)	0.30416 (18)	0.0849 (9)	
O4	0.5658 (2)	0.84529 (16)	0.48997 (14)	0.0683 (7)	
C19'	0.0535 (17)	0.7807 (15)	0.2940 (10)	0.123 (8)	0.49
H19D	0.0884	0.7667	0.3504	0.184*	0.49
H19E	−0.0025	0.7369	0.2658	0.184*	0.49
H19F	0.0041	0.8311	0.2885	0.184*	0.49
C18'	0.160 (2)	0.7920 (11)	0.2596 (11)	0.099 (7)	0.49
H18C	0.1275	0.8040	0.2020	0.119*	0.49
H18D	0.2153	0.7430	0.2675	0.119*	0.49
C24'	0.8165 (13)	0.5613 (7)	0.3901 (10)	0.064 (4)	0.29
H24C	0.7551	0.5582	0.3359	0.077*	0.29
H24D	0.7973	0.5170	0.4226	0.077*	0.29
C25'	0.9573 (15)	0.5544 (12)	0.3871 (14)	0.114 (8)	0.29
H25D	0.9900	0.6086	0.3813	0.171*	0.29
H25E	0.9590	0.5206	0.3418	0.171*	0.29
H25F	1.0110	0.5296	0.4364	0.171*	0.29

### Atomic displacement parameters (Å^2^)

**Table:**

	*U*^11^	*U*^22^	*U*^33^	*U*^12^	*U*^13^	*U*^23^
C1	0.120 (4)	0.103 (4)	0.069 (3)	0.023 (3)	0.010 (3)	0.017 (3)
C2	0.106 (3)	0.090 (3)	0.058 (2)	0.009 (3)	0.037 (2)	0.017 (2)
C3	0.068 (2)	0.067 (2)	0.053 (2)	0.007 (2)	0.0323 (19)	0.0092 (18)
C4	0.0394 (17)	0.057 (2)	0.0489 (18)	0.0009 (15)	0.0223 (14)	0.0042 (15)
C5	0.0530 (19)	0.054 (2)	0.0459 (18)	0.0040 (16)	0.0219 (15)	0.0022 (15)
C6	0.051 (2)	0.114 (4)	0.085 (3)	−0.023 (2)	0.016 (2)	0.016 (3)
C7	0.0445 (18)	0.0460 (19)	0.0444 (17)	−0.0024 (15)	0.0150 (14)	−0.0006 (14)
C8	0.0415 (17)	0.0505 (19)	0.0354 (15)	0.0011 (14)	0.0156 (13)	0.0014 (13)
C9	0.0433 (17)	0.0491 (19)	0.0426 (17)	0.0016 (14)	0.0202 (14)	0.0013 (15)
C10	0.060 (2)	0.055 (2)	0.0413 (17)	0.0009 (17)	0.0167 (15)	0.0000 (16)
C11	0.072 (2)	0.072 (3)	0.058 (2)	−0.015 (2)	0.0188 (19)	−0.016 (2)
C12	0.081 (3)	0.046 (2)	0.087 (3)	−0.013 (2)	0.041 (2)	−0.014 (2)
C13	0.074 (2)	0.052 (2)	0.070 (2)	0.0044 (19)	0.027 (2)	0.0065 (19)
C14	0.0527 (19)	0.052 (2)	0.0491 (19)	0.0011 (16)	0.0172 (15)	0.0026 (16)
C15	0.0468 (18)	0.0497 (19)	0.0441 (17)	−0.0018 (15)	0.0213 (14)	0.0034 (15)
C16	0.0428 (18)	0.056 (2)	0.056 (2)	−0.0021 (17)	0.0206 (15)	0.0040 (17)
C17	0.049 (2)	0.066 (3)	0.0500 (19)	−0.0080 (18)	0.0183 (15)	−0.0022 (17)
C20	0.0542 (19)	0.049 (2)	0.0430 (17)	−0.0028 (16)	0.0180 (15)	0.0033 (15)
C21	0.056 (2)	0.047 (2)	0.0459 (18)	−0.0028 (16)	0.0180 (15)	0.0041 (15)
C22	0.074 (2)	0.055 (2)	0.052 (2)	0.0086 (18)	0.0182 (18)	0.0001 (17)
C23	0.140 (4)	0.065 (3)	0.074 (3)	0.039 (3)	0.034 (3)	0.001 (2)
C18	0.057 (7)	0.081 (8)	0.099 (11)	−0.032 (6)	0.031 (7)	−0.017 (7)
C19	0.083 (10)	0.159 (17)	0.20 (2)	−0.053 (9)	−0.012 (13)	−0.026 (17)
C24	0.096 (7)	0.066 (5)	0.106 (6)	0.006 (5)	0.030 (6)	−0.002 (4)
C25	0.169 (9)	0.088 (6)	0.144 (8)	−0.016 (6)	0.076 (6)	−0.025 (6)
C26	0.147 (4)	0.079 (3)	0.053 (2)	0.015 (3)	0.034 (3)	0.016 (2)
Cl1	0.0643 (6)	0.0700 (6)	0.0486 (5)	−0.0069 (4)	0.0125 (4)	−0.0022 (4)
Cl2	0.1617 (14)	0.0646 (8)	0.1425 (13)	−0.0441 (8)	0.0452 (10)	−0.0271 (8)
Cl3	0.0878 (7)	0.0770 (7)	0.0490 (5)	0.0122 (5)	0.0263 (5)	−0.0028 (5)
N1	0.068 (2)	0.085 (2)	0.064 (2)	−0.0108 (18)	0.0332 (17)	0.0137 (18)
N2	0.0485 (16)	0.075 (2)	0.0597 (18)	−0.0044 (15)	0.0225 (14)	0.0099 (16)
N3	0.0416 (14)	0.0435 (15)	0.0420 (13)	−0.0004 (12)	0.0172 (11)	0.0034 (12)
N4	0.0542 (19)	0.074 (2)	0.094 (2)	0.0064 (17)	0.0310 (17)	0.0015 (19)
N5	0.154 (4)	0.066 (2)	0.073 (2)	0.041 (2)	0.027 (2)	0.0145 (19)
N6	0.098 (2)	0.0534 (19)	0.0487 (17)	0.0085 (17)	0.0219 (16)	0.0094 (15)
O1	0.0428 (13)	0.0702 (16)	0.0600 (14)	0.0027 (11)	0.0069 (11)	0.0128 (12)
O2	0.0635 (16)	0.0582 (17)	0.0903 (19)	−0.0093 (14)	0.0278 (14)	−0.0143 (14)
O3	0.0497 (15)	0.082 (2)	0.120 (2)	−0.0187 (14)	0.0214 (15)	−0.0215 (18)
O4	0.0842 (17)	0.0763 (18)	0.0597 (15)	0.0154 (14)	0.0445 (14)	0.0172 (13)
C19'	0.134 (18)	0.129 (14)	0.128 (14)	−0.063 (14)	0.072 (12)	−0.039 (12)
C18'	0.074 (10)	0.100 (10)	0.122 (15)	−0.028 (7)	0.027 (10)	−0.012 (10)
C24'	0.067 (10)	0.043 (9)	0.081 (10)	−0.003 (8)	0.020 (9)	−0.007 (8)
C25'	0.097 (15)	0.124 (18)	0.142 (17)	0.032 (13)	0.068 (13)	−0.024 (14)

### Geometric parameters (Å, °)

**Table:**

C1—C2	1.505 (6)	C20—C21	1.460 (5)
C1—H1A	0.9600	C21—N6	1.353 (4)
C1—H1B	0.9600	C21—C22	1.381 (5)
C1—H1C	0.9600	C22—C23	1.382 (6)
C2—C3	1.499 (5)	C22—Cl3	1.710 (4)
C2—H2A	0.9700	C23—N5	1.348 (5)
C2—H2B	0.9700	C23—C24'	1.484 (9)
C3—N1	1.343 (5)	C23—C24	1.542 (7)
C3—C5	1.387 (5)	C18—C19	1.465 (10)
C4—N2	1.358 (4)	C18—O3	1.475 (8)
C4—C5	1.383 (4)	C18—H18A	0.9700
C4—C7	1.476 (4)	C18—H18B	0.9700
C5—Cl1	1.716 (3)	C19—H19A	0.9600
C6—N2	1.465 (5)	C19—H19B	0.9600
C6—H6A	0.9600	C19—H19C	0.9600
C6—H6B	0.9600	C24—C25	1.481 (7)
C6—H6C	0.9600	C24—H24A	0.9700
C7—O1	1.202 (3)	C24—H24B	0.9700
C7—N3	1.423 (4)	C25—H25A	0.9600
C8—C15	1.356 (4)	C25—H25B	0.9600
C8—N3	1.415 (4)	C25—H25C	0.9600
C8—C9	1.475 (4)	C26—N6	1.457 (5)
C9—C14	1.387 (4)	C26—H26A	0.9600
C9—C10	1.395 (4)	C26—H26B	0.9600
C10—C11	1.376 (5)	C26—H26C	0.9600
C10—H10	0.9300	N1—N2	1.349 (4)
C11—C12	1.370 (6)	N5—N6	1.345 (4)
C11—H11	0.9300	O3—C18'	1.458 (9)
C12—C13	1.367 (5)	C19'—C18'	1.456 (10)
C12—Cl2	1.752 (4)	C19'—H19D	0.9600
C13—C14	1.379 (5)	C19'—H19E	0.9600
C13—H13	0.9300	C19'—H19F	0.9600
C14—H14	0.9300	C18'—H18C	0.9700
C15—C16	1.436 (5)	C18'—H18D	0.9700
C15—C17	1.491 (5)	C24'—C25'	1.531 (10)
C16—N4	1.139 (4)	C24'—H24C	0.9700
C17—O2	1.196 (4)	C24'—H24D	0.9700
C17—O3	1.323 (4)	C25'—H25D	0.9600
C20—O4	1.197 (4)	C25'—H25E	0.9600
C20—N3	1.444 (4)	C25'—H25F	0.9600
			
C2—C1—H1A	109.5	C16—C15—C17	116.7 (3)
C2—C1—H1B	109.5	N4—C16—C15	178.5 (4)
H1A—C1—H1B	109.5	O2—C17—O3	123.8 (3)
C2—C1—H1C	109.5	O2—C17—C15	125.5 (3)
H1A—C1—H1C	109.5	O3—C17—C15	110.7 (3)
H1B—C1—H1C	109.5	O4—C20—N3	119.2 (3)
C3—C2—C1	113.1 (3)	O4—C20—C21	123.3 (3)
C3—C2—H2A	109.0	N3—C20—C21	117.5 (3)
C1—C2—H2A	109.0	N6—C21—C22	105.6 (3)
C3—C2—H2B	109.0	N6—C21—C20	120.0 (3)
C1—C2—H2B	109.0	C22—C21—C20	134.3 (3)
H2A—C2—H2B	107.8	C21—C22—C23	107.1 (3)
N1—C3—C5	110.1 (3)	C21—C22—Cl3	127.6 (3)
N1—C3—C2	120.5 (3)	C22—C21—C20	134.3 (3)
C5—C3—C2	129.4 (4)	C21—C22—C23	107.1 (3)
N2—C4—C5	105.2 (3)	C21—C22—Cl3	127.6 (3)
N2—C4—C7	120.0 (3)	C23—C22—Cl3	125.2 (3)
C5—C4—C7	134.7 (3)	N5—C23—C22	109.3 (3)
C4—C5—C3	106.8 (3)	N5—C23—C24'	116.2 (8)
C4—C5—Cl1	128.4 (3)	C22—C23—C24'	128.4 (8)
C3—C5—Cl1	124.7 (3)	N5—C23—C24	122.2 (5)
N2—C6—H6A	109.5	C22—C23—C24	127.2 (5)
N2—C6—H6B	109.5	C19—C18—O3	102.4 (8)
H6A—C6—H6B	109.5	C19—C18—H18A	111.3
N2—C6—H6C	109.5	O3—C18—H18A	111.3
H6A—C6—H6C	109.5	C19—C18—H18B	111.3
H6B—C6—H6C	109.5	O3—C18—H18B	111.3
O1—C7—N3	121.5 (3)	H18A—C18—H18B	109.2
O1—C7—C4	123.4 (3)	C25—C24—C23	105.6 (6)
N3—C7—C4	115.1 (3)	C25—C24—H24A	110.6
C15—C8—N3	121.8 (3)	C23—C24—H24A	110.6
C15—C8—C9	122.7 (3)	C25—C24—H24B	110.6
N3—C8—C9	115.3 (2)	C23—C24—H24B	110.6
C14—C9—C10	119.2 (3)	H24A—C24—H24B	108.7
C14—C9—C8	121.5 (3)	N6—C26—H26A	109.5
C10—C9—C8	119.3 (3)	N6—C26—H26B	109.5
C11—C10—C9	120.3 (3)	H26A—C26—H26B	109.5
C11—C10—H10	119.9	N6—C26—H26C	109.5
C9—C10—H10	119.9	H26A—C26—H26C	109.5
C12—C11—C10	118.8 (3)	H26B—C26—H26C	109.5
C12—C11—H11	120.6	C3—N1—N2	105.4 (3)
C10—C11—H11	120.6	N1—N2—C4	112.4 (3)
C13—C12—C11	122.5 (3)	N1—N2—C6	118.0 (3)
C13—C12—Cl2	118.8 (3)	C4—N2—C6	129.4 (3)
C11—C12—Cl2	118.7 (3)	C8—N3—C7	118.8 (2)
C12—C13—C14	118.6 (4)	C8—N3—C20	117.9 (2)
C12—C13—H13	120.7	C7—N3—C20	119.2 (2)
C14—C13—H13	120.7	N6—N5—C23	106.1 (3)
C13—C14—C9	120.6 (3)	N5—N6—C21	111.9 (3)
C13—C14—H14	119.7	N5—N6—C26	118.7 (3)
C9—C14—H14	119.7	C21—N6—C26	129.4 (3)
C8—C15—C16	119.0 (3)	C17—O3—C18'	117.5 (10)
C8—C15—C17	124.3 (3)	C17—O3—C18	114.0 (8)
C16—C15—C17	116.7 (3)	C18'—C19'—H19D	109.5
N4—C16—C15	178.5 (4)	C18'—C19'—H19E	109.5
O2—C17—O3	123.8 (3)	H19D—C19'—H19E	109.5
O2—C17—C15	125.5 (3)	C18'—C19'—H19F	109.5
O3—C17—C15	110.7 (3)	H19D—C19'—H19F	109.5
O4—C20—N3	119.2 (3)	H19E—C19'—H19F	109.5
O4—C20—C21	123.3 (3)	C19'—C18'—O3	103.9 (9)
N3—C20—C21	117.5 (3)	C19'—C18'—H18C	111.0
N6—C21—C22	105.6 (3)	O3—C18'—H18C	111.0
N6—C21—C20	120.0 (3)	C19'—C18'—H18D	111.0
C22—C21—C20	134.3 (3)	O3—C18'—H18D	111.0
C21—C22—C23	107.1 (3)	H18C—C18'—H18D	109.0
C21—C22—Cl3	127.6 (3)	C23—C24'—C25'	106.0 (8)
C10—C11—H11	120.6	C23—C24'—H24C	110.5
C13—C12—C11	122.5 (3)	C25'—C24'—H24C	110.5
C13—C12—Cl2	118.8 (3)	C23—C24'—H24D	110.5
C11—C12—Cl2	118.7 (3)	C25'—C24'—H24D	110.5
C12—C13—C14	118.6 (4)	H24C—C24'—H24D	108.7
C12—C13—H13	120.7	C24'—C25'—H25D	109.5
C14—C13—H13	120.7	C24'—C25'—H25E	109.5
C13—C14—C9	120.6 (3)	H25D—C25'—H25E	109.5
C13—C14—H14	119.7	C24'—C25'—H25F	109.5
C9—C14—H14	119.7	H25D—C25'—H25F	109.5
C8—C15—C16	119.0 (3)	H25E—C25'—H25F	109.5
C8—C15—C17	124.3 (3)		
			
C1—C2—C3—N1	−107.3 (5)	Cl3—C22—C23—C24'	−26.2 (10)
C1—C2—C3—C5	72.6 (6)	C21—C22—C23—C24	−166.6 (5)
N2—C4—C5—C3	0.2 (4)	Cl3—C22—C23—C24	16.2 (8)
C7—C4—C5—C3	−176.8 (3)	N5—C23—C24—C25	76.6 (8)
N2—C4—C5—Cl1	178.8 (2)	C22—C23—C24—C25	−118.3 (7)
C7—C4—C5—Cl1	1.8 (6)	C24'—C23—C24—C25	−12.9 (14)
N1—C3—C5—C4	−1.2 (4)	C5—C3—N1—N2	1.6 (4)
C2—C3—C5—C4	178.9 (4)	C2—C3—N1—N2	−178.4 (3)
N1—C3—C5—Cl1	−179.8 (3)	C3—N1—N2—C4	−1.6 (4)
C2—C3—C5—Cl1	0.2 (6)	C3—N1—N2—C6	−176.7 (3)
N2—C4—C7—O1	−33.8 (5)	C5—C4—N2—N1	0.8 (4)
C5—C4—C7—O1	142.9 (4)	C7—C4—N2—N1	178.4 (3)
N2—C4—C7—N3	145.1 (3)	C5—C4—N2—C6	175.3 (4)
C5—C4—C7—N3	−38.2 (5)	C7—C4—N2—C6	−7.1 (5)
C15—C8—C9—C14	−51.9 (4)	C15—C8—N3—C7	149.4 (3)
N3—C8—C9—C14	133.3 (3)	C9—C8—N3—C7	−35.7 (4)
C15—C8—C9—C10	130.3 (3)	C15—C8—N3—C20	−53.3 (4)
N3—C8—C9—C10	−44.5 (4)	C9—C8—N3—C20	121.6 (3)
C14—C9—C10—C11	0.7 (5)	O1—C7—N3—C8	140.0 (3)
C8—C9—C10—C11	178.5 (3)	C4—C7—N3—C8	−38.9 (4)
C9—C10—C11—C12	−1.4 (5)	O1—C7—N3—C20	−17.0 (4)
C10—C11—C12—C13	1.7 (6)	C4—C7—N3—C20	164.1 (3)
C10—C11—C12—Cl2	−178.5 (3)	O4—C20—N3—C8	−16.6 (4)
C11—C12—C13—C14	−1.3 (6)	C21—C20—N3—C8	163.2 (3)
Cl2—C12—C13—C14	178.9 (3)	O4—C20—N3—C7	140.6 (3)
C12—C13—C14—C9	0.5 (5)	C21—C20—N3—C7	−39.6 (4)
C10—C9—C14—C13	−0.2 (5)	C22—C23—N5—N6	0.0 (6)
C8—C9—C14—C13	−178.0 (3)	C24'—C23—N5—N6	−154.8 (7)
N3—C8—C15—C16	170.3 (3)	C24—C23—N5—N6	167.5 (5)
C9—C8—C15—C16	−4.3 (4)	C23—N5—N6—C21	−0.2 (5)
N3—C8—C15—C17	−9.9 (5)	C23—N5—N6—C26	−179.5 (4)
C9—C8—C15—C17	175.6 (3)	C22—C21—N6—N5	0.2 (4)
C8—C15—C17—O2	−3.5 (5)	C20—C21—N6—N5	178.0 (3)
C16—C15—C17—O2	176.4 (3)	C22—C21—N6—C26	179.5 (4)
C8—C15—C17—O3	177.0 (3)	C20—C21—N6—C26	−2.8 (6)
C16—C15—C17—O3	−3.1 (4)	O2—C17—O3—C18'	−14.9 (10)
O4—C20—C21—N6	−27.8 (5)	C15—C17—O3—C18'	164.7 (9)
N3—C20—C21—N6	152.4 (3)	O2—C17—O3—C18	9.9 (9)
O4—C20—C21—C22	149.1 (4)	C15—C17—O3—C18	−170.5 (8)
N3—C20—C21—C22	−30.7 (5)	C19—C18—O3—C17	−164.7 (12)
N6—C21—C22—C23	−0.2 (4)	C19—C18—O3—C18'	−60 (4)
C20—C21—C22—C23	−177.4 (4)	C17—O3—C18'—C19'	142.5 (12)
N6—C21—C22—Cl3	176.9 (3)	C18—O3—C18'—C19'	56 (4)
C20—C21—C22—Cl3	−0.3 (6)	N5—C23—C24'—C25'	−103.8 (14)
C21—C22—C23—N5	0.1 (6)	C22—C23—C24'—C25'	107.0 (14)
Cl3—C22—C23—N5	−177.1 (3)	C24—C23—C24'—C25'	5.7 (12)
C21—C22—C23—C24'	151.0 (8)		

### Hydrogen-bond geometry (Å, °)

**Table:**

*D*—H···*A*	*D*—H	H···*A*	*D*···*A*	*D*—H···*A*
C13—H13···O2^i^	0.93	2.59	3.420 (5)	149

Symmetry codes: (i) −*x*+1, *y*+1/2, −*z*+1/2.
